# Case Report: Local anaesthetic systemic toxicity secondary to presumed thoracostomy tube migration through the aortic hiatus in a dog

**DOI:** 10.3389/fvets.2026.1747858

**Published:** 2026-02-19

**Authors:** Wilmer A. Lopez, Caitlin M. Bowen, Lauren A. Cochran, Denae N. Campanale

**Affiliations:** Bluepearl Pet Hospital, Tampa, FL, United States

**Keywords:** aortic hiatus, complication, local anaestetic, migration, thoracostomy tube, toxicicity

## Abstract

A 9-year-old neutered male Golden Retriever was presented in cardiac tamponade. Initial stabilization involved pericardiocentesis, and through exclusion, a diagnosis of idiopathic pericardial effusion was made. The patient experienced multiple episodes of recurrent cardiac tamponade, and a subtotal pericardiectomy was ultimately performed. A thoracostomy tube was placed intraoperatively to aid in postoperative management and analgesia (intrapleural bupivacaine, 1.5 mg/kg q6hr). Approximately one hour after the third bupivacaine dose, the patient experienced acute onset of tachycardia, hypoxemia, and dull mentation. Repeat thoracic radiographs suggested migration of the thoracostomy tube through the aortic hiatus into the abdomen, followed by the suspicion of local anaesthetic systemic toxicity. The thoracostomy tube was subsequently removed and the patient was treated with intravenous intralipid emulsion. The patient survived without any long-term complications. This case highlights a previously unreported complication of presumptive thoracostomy tube migration into the abdomen of a dog via the aortic hiatus. It also describes peri-aortic administration of bupivacaine resulting in suspected local anaesthetic systemic toxicity. This underscores the importance of close patient monitoring following thoracostomy tube placement, thorough client education regarding potential complications, and repeat thoracic imaging in any patient with a thoracostomy tube that develops respiratory or cardiovascular compromise.

## Introduction

Pericardial effusion (PCE) in dogs is commonly caused by neoplasia, idiopathic pericarditis, trauma, and less commonly by infectious, metabolic, or immune-mediated diseases (1–3). Idiopathic pericardial effusion is a diagnosis of exclusion that is presumed when no underlying cause is identified after thorough evaluation including echocardiography, computed tomography (CT), and pericardial histopathology. Idiopathic pericardial effusion is most prevalent in large-breed dogs, especially Golden Retrievers (1, 2).

Treatment options for pericardial effusion depend on the underlying cause and clinical presentation. For dogs with significant hemodynamic compromise, pericardiocentesis is the initial intervention recommended for hemodynamic stabilization (1–3). Subtotal pericardiectomy is the preferred surgical treatment to minimize long-term recurrence of pericardial effusion (2–6). Surgical approaches for subtotal pericardiectomy include thoracotomy, thoracoscopy, or transdiaphragmatic approaches, all with similar efficacy; however, subtotal pericardiectomy via thoracotomy offers superior long-term outcomes for idiopathic effusion (3–6). Tube thoracostomy is indicated in dogs with pericardial effusion when there is concurrent significant pleural effusion that requires active drainage, and when intraoperative or postoperative management of pleural fluid, air, or both is necessary (1, 3). Thoracostomy tubes are also used perioperatively to aid in analgesia via intrapleural administration of local anaesthetic agents (7).

This case report presents a previously unreported occurrence of thoracostomy tube migration presumably through the aortic hiatus into the abdomen of a dog following subtotal pericardiectomy for idiopathic pericarditis. This highlights a potential complication of chest tube placement in this population of patients, and documents the significance of routine diagnostic imaging, patient monitoring, and appropriate analgesic selection following thoracostomy tube placement.

## Case summary

An approximately 9-year-old, 55.0-kilogram, neutered male Golden Retriever presented to a veterinary urgent care facility with a two-week history of syncope during routine walks and recent onset of dyspnea. On initial presentation, the patient was panting and physical examination revealed tachycardia, marked obesity (body condition score 9/9), and abdominal distension. Femoral pulses were difficult to palpate.

A complete blood count (CBC) revealed several abnormalities, including reticulocytosis without anemia (148.6 K/μL; Reference Interval (RI) 10–110 K/μL), and leukocytosis (21.76 K/μL; RI 5.05–16.76 K/μL) with neutrophilia (14.06 K/μL; RI 2.95–11.64 K/μL) and lymphocytosis (6.46 K/μL; RI 1.05–5.10 K/μL). The mean platelet volume (MPV) of 14.8 fL (RI 8.7–13.2 fL) and plateletcrit at 0.53% (RI 0.14–0.46%) were also elevated. A serum biochemistry panel revealed mild hyperglycemia (154 mg/dL; RI 70–143 mg/dL) but was otherwise unremarkable.

Point-of-care ultrasound (POCUS) of the abdomen revealed moderate to severe peritoneal effusion, most prominent in the cranial abdomen in the hepatosplenic quadrant. Thoracic POCUS revealed a moderate volume of pericardial effusion (PCE), with no pleural effusion observed. Abdominocentesis yielded grossly sero-sanguineous fluid. The patient was then transferred as an emergency to a referral veterinary hospital for further evaluation and treatment.

At the referral center, PCV/TS, lactate, and blood glucose via glucometer were within normal limits. A systolic blood pressure obtained via Doppler measured 160 mmHg (RI 100–145 mmHg). Coagulation parameters [prothrombin time (PT) and partial thromboplastin time (PTT)] were normal. Repeat thoracic and abdominal POCUS revealed the same findings as previously. A pericardiocentesis was performed and 500 mL of grossly hemorrhagic effusion was removed from the pericardial space and submitted for cytology and fluid analysis. Echocardiography revealed no cardiac masses nor significant structural cardiac abnormalities. Abdominal ultrasound showed a hyperechoic and mottled region in the right liver accompanied by a single anechoic cystic structure. The remainder of the liver was diffusely mildly mottled. Moderate peritoneal effusion was noted. The remainder of the study was unremarkable. Ultrasound-guided abdominocentesis and fine-needle aspirates (FNA) of the mottled right liver lobe were obtained routinely. The pericardial effusion was consistent with a hemorrhagic effusion. The abdominal effusion was characterized as a modified transudate. Liver cytology revealed mild hepatocellular atypia consistent with reactive or hyperplastic changes, mild glycogen/water accumulation, and an increased percentage of neutrophils. No infectious or neoplastic etiologies were noted.

The patient was discharged without medications however re-presented twice approximately 1 week and 1 month after initial discharge, each time for concerns of cardiac tamponade and requiring repeated pericardiocenteses. The pericardial effusion was presumed idiopathic. A subtotal pericardiectomy was subsequently performed via a right fifth intercostal thoracotomy. At the time of the procedure the patient weighed 49.7-kilograms. Premedication consisted of methadone (0.2 mg/kg) and midazolam (0.2 mg/kg IV). Anaesthesia was induced with propofol IV to effect and maintained with fentanyl (10–30 μg/kg/h IV), ketamine (5–20 μg/kg/min IV), lidocaine (25–50 μg/kg/min IV), and isoflurane (0.5–1.0% volume percent). No evidence of neoplasia was noted at time of surgery. No anaesthetic or perioperative complications were observed, and recovery was smooth.

**Figure 1:**
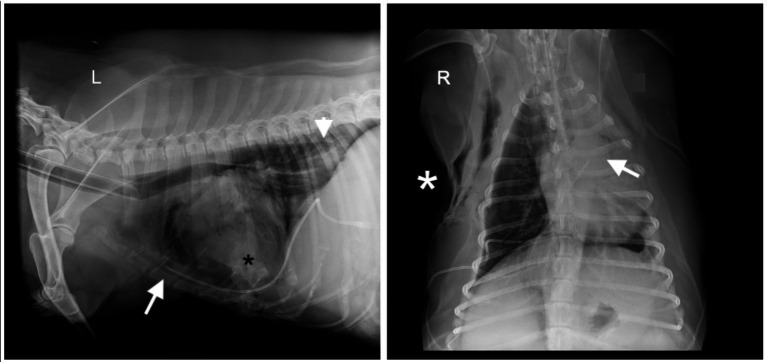
Left lateral (L) and right ventrodorsal (R) thoracic radiographs of a 9-year-old, 55.0 kg, neutered male Golden Retriever obtained immediately following subtotal pericardiectomy. A radiopaque thoracostomy tube (MILA Chest Tube; MILA International Inc.) placed through the right eighth intercostal space is visible within the pleural cavity (white arrow). Moderate volumes of fluid and gas are present within the pleural space and possibly the mediastinum (black asterisk). The esophagus is moderately gas-distended with a small amount of caudal fluid (white arrowhead). The lungs are diffusely underinflated with mild, diffuse, unstructured pulmonary opacity. Moderate subcutaneous emphysema along the right thoracic body wall is consistent with recent surgery (white asterisk).

**Figure 2:**
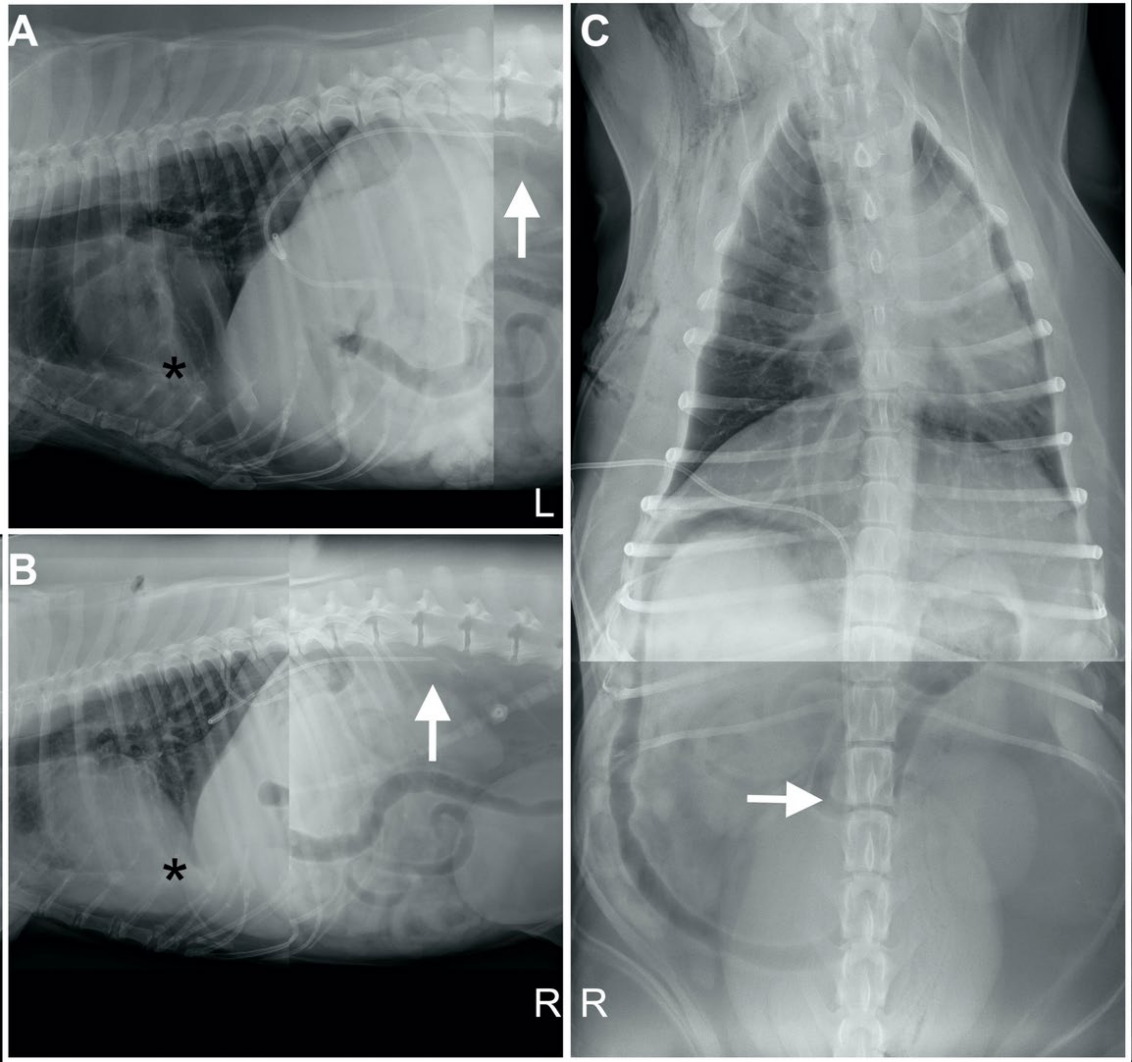
Left lateral **(A)**, right lateral **(B)**, and ventrodorsal **(C)** composite thoracic and abdominal radiographic images of a 9-year-old, 55.0 kg, neutered male Golden Retriever approximately 24 h post-subtotal pericardiectomy. The radiopaque thoracostomy tube (MILA Chest Tube; MILA International Inc.), inserted in the right eighth intercostal space, is displaced caudally from its prior and expected position (Figure 1). The tube now summates with the caudal thorax and dorsal abdomen, with the tip terminating ventral to the second lumbar vertebra (L2) on midline. Mild, wispy fluid–soft tissue opacity is present within the retroperitoneal space surrounding the catheter tip (white arrow). Pleural abnormalities have improved, with only small residual volumes of fluid and gas (black asterisk).

A thoracostomy tube (MILA Chest Tube, MILA International Inc.) was placed in the right hemithorax. No trochar was utilized, and the tube was placed intraoperatively prior to closure allowing for visualization of the tube was within the pleural space running along the sterum. Post-operative radiographs also confirmed appropriate placement (Figure 1). The patient remained in hospital for post-operative monitoring. Following surgery, the patient was receiving intrapleural bupivacaine at 1.5 mg/kg every 6 h, undiluted followed with 5 mL of sterile saline, through the chest tube for analgesic purposes. Overnight, he remained stable and comfortable with no arrhythmias noted on telemetry. However, approximately 1 h after the third bupivacaine dose, the patient acutely developed tachycardia (212 bpm) with hypoxemia (saturation of peripheral oxygenation via pulse oximetry: 89%), dull mentation, and injected mucous membranes with a brisk capillary refill time (<1 s). No dose or administration errors were observed. Blood pressure measurements remained normal (130 mmHg), and direct pupillary light reflexes remained intact. Flow by oxygen was given, and oxygen saturation normalized. Repeat thoracic radiographs revealed that the thoracostomy tube had either kinked or flipped and was tracking dorsally. The proximal aspect (tip) of the tube was in the cranial-to-mid abdomen along the dorsal midline, suggesting that the chest tube had migrated along the aortic hiatus into the peritoneal or retroperitoneal space (Figure 2).

It was suspected that the bupivacaine had been inadvertently instilled in the abdomen, near the aorta, leading to systemic bupivacaine toxicity. The chest tube was removed approximately 1 h following radiographs, and intravenous intralipid emulsion (ILE) was initiated, using lean body weight of 35 kg, 1.5 mL/kg bolus over 15 min, followed by continuous rate infusion of 0.25 mL/kg/min for about 30 min, approximately 3 h following onset of clinical signs. There was no appreciable change in pain score. Heart rate improved some within the hour of removal of the chest tube (170 bpm). However, following ILE treatment, the patient showed marked clinical improvement within the hour; oxygenation normalized without supplemental oxygen, heart rate decreased though remained mildly elevated (134 bpm), dyspnea resolved, CRT normalized, and mentation significantly improved. The patient was discharged 2 days later.

Histopathology of the excised pericardial tissue showed chronic, mild lymphoplasmacytic infiltrate and chronic hemorrhage consistent with exuberant fibroplasia; supporting a clinical diagnosis of chronic idiopathic pericarditis. Following surgery, no episodes of syncope or dyspnea recurred. The patient survived the complications described and had no known related long-term consequences. The patient re-presented approximately 15 months later to the referral hospital for a partial cranial-cruciate ligament tear and underwent a tibial plateau leveling osteotomy (timeline of events summarized in Table 1).

**Table 1:** Timeline of clinical presentation, diagnostics, treatments, and outcomes in a 9-year-old neutered male Golden Retriever with idiopathic pericardial effusion.

Time frame	Event	Findings/actions/outcomes
2 weeks before presentation	Onset of clinical signs	Owner noted syncope during routine walks and recent onset of dyspnea.
Day 0: Initial presentation (urgent care)	Presentation to urgent care	9-year-old, 55 kg neutered male Golden Retriever; panting, tachycardic, obese (BCS 9/9), abdominal distension; weak femoral pulses.
	Diagnostics: CBC	Reticulocytosis without anemia (148.6 K/μL ↑), leukocytosis (21.76 K/μL ↑), neutrophilia (14.06 K/μL ↑), lymphocytosis (6.46 K/μL ↑), ↑MPV (14.8 fL), ↑plateletcrit (0.53%).
	Diagnostics: Serum biochemistry	Mild hyperglycemia (154 mg/dL), otherwise unremarkable.
	POCUS (thoracic/abdominal)	Moderate-to-severe peritoneal effusion; moderate pericardial effusion (no pleural effusion).
	Abdominocentesis	Grossly clear, pink-tinged fluid obtained.
	Action	Patient transferred to referral emergency/specialty hospital.
Day 1: Referral hospital evaluation	Initial assessment	PCV/TS, lactate, glucose all WNL; BP 160 mmHg (↑); PT/PTT normal.
	Repeat POCUS	Findings unchanged; pericardial and peritoneal effusion persist.
	Pericardiocentesis performed	500 mL hemorrhagic effusion removed; submitted for cytology/fluid analysis.
	Echocardiogram	No cardiac masses or significant structural abnormalities.
	Abdominal ultrasound	Mildly mottled, hyperechoic right liver with anechoic cystic structure; moderate peritoneal effusion.
	FNA of liver and effusion	No complications; liver cytology: mild reactive changes, glycogen/water accumulation, ↑neutrophils; effusion = modified transudate; pericardial effusion = hemorrhagic, noninfectious, nonneoplastic.
	Diagnosis/impression	Idiopathic pericardial effusion suspected.
	Discharge	No medications prescribed.
~1 week post-discharge	Re-presentation #1	Recurrent pericardial effusion/cardiac tamponade; repeat pericardiocentesis performed.
~1 month post-discharge	Re-presentation #2	Second recurrence of pericardial effusionrepeat pericardiocentesis.
Subsequent intervention	Subtotal pericardiectomy	Performed via right fifth intercostal thoracotomy; MILA thoracostomy tube placed without trochar under direct visualization.
Post-op day 0	Recovery	Uneventful anaesthesia recovery; stable overnight, no arrhythmias on telemetry; radiographs confirmed correct tube placement.
Post-op day 1 (~1 h after third bupivicaine dose)	Acute deterioration	Developed supraventricular tachycardia (212 bpm), SpO₂ 89%, dull mentation, injected MM, brisk CRT; BP 130 mmHg (normal).
	Diagnostics	Radiographs: chest tube migrated via aortic hiatus into abdomen (kinked/flipped).
	Suspected cause	Intrapleural bupivacaine (1.5 mg/kg q6h) likely infused into aortic perivascular space, causing systemic bupivacaine toxicity.
	Treatment	Chest tube removed; IV intralipid therapy initiated (1.5 mL/kg bolus → 0.25 mL/kg/min CRI × 30 min).
	Response	Rapid improvement (within 1 h): HR ↓ to 134 bpm, normal oxygenation, dyspnea resolved, mentation normalized.
Post-op day 3 (discharge)	Discharged home	Stable, no further complications.
Histopathology results	Pericardium	Chronic mild lymphoplasmacytic infiltrate and hemorrhage with fibroplasia → chronic idiopathic pericarditis.
Following months	Outcome	No recurrence of syncope or dyspnea; full recovery from post-op complications.
~15 months post-surgery	Unrelated condition	Re-presented for partial cranial cruciate ligament tear; underwent tibial plateau leveling osteotomy (TPLO).

The patient experienced recurrent cardiac tamponade requiring subtotal pericardiectomy. Postoperatively, chest tube migration led to inadvertent bupivacaine infusion and systemic toxicity, successfully treated with intravenous intralipid therapy. Histopathology confirmed chronic idiopathic pericarditis, and the patient recovered fully with no recurrence of pericardial effusion.

## Discussion

To the authors’ knowledge, this is the first reported case in the veterinary and human literature of presumed thoracostomy tube migration into the abdomen via the aortic hiatus.

The pericardium is a double-layered sac that encloses the heart and the roots of the great vessels, including the aorta (8, 9). Within the thorax, the aorta is divided anatomically into the ascending aorta, aortic arch, and descending aorta. The ascending aorta originates from the left ventricle, courses cranially, and curves dorsally and to the left to form the aortic arch before continuing caudally as the descending aorta along the left side of the thorax. The aorta then passes through the diaphragm via the aortic hiatus to enter the abdomen. The aortic hiatus is bound dorsally by the vertebral column, laterally by the crura of the diaphragm, and ventrally by the median arcuate ligament (9, 10). Unlike the esophageal hiatus, which is reinforced by a membranous structure, the aortic hiatus lacks a dedicated fascial or membranous covering. Its structural integrity is instead provided by the crura and their close apposition (10).

Conceptually, migration of a chest tube through the aortic hiatus is exceedingly rare and considered anatomically unlikely given standard insertion techniques and the natural barriers involved (11). However, if malposition or migration into the aortic hiatus occurs, catastrophic complications such as aortic perforation, massive hemorrhage, and acute hemodynamic collapse are possible (11). Previous human case reports describe chest tubes traversing the diaphragm and entering the abdominal cavity, often associated with abnormal diaphragmatic anatomy or misidentification of the diaphragm during placement. For example, abdominal tube placement has been reported in a patient with diaphragmatic paralysis, where failure to recognize elevation of the diaphragm, along with use of a trocar, allowed the tube to traverse the diaphragm (12). There are also rare reported cases in people of chest tube migration into the esophageal hiatus or esophagus, specifically in the context of esophageal perforation (13). Such cases highlight the role of the diaphragm as the principal barrier preventing entry into the abdomen during thoracostomy tube placement. Based on these case reports, populations at increased risk for chest tube malposition include patients with congenital diaphragmatic defects, esophageal perforation, diaphragmatic paralysis, iatrogenic injury, or ascites associated with liver disease (11–14).

The present case describes a unique scenario, in which the chest tube that was initially positioned appropriately (Figure 1) then subsequently migrated into an aberrant location (Figure 2). Numerous factors contribute to chest tube migration, with the most relevant to this case being patient body habitus and agitation. In human medicine, obese patients, patients who lie on their tubes, and those experiencing restlessness, frequent repositioning, or transport, are at higher risk for tube kinking, levering, and migration (11, 15). In this case, the patient was markedly overweight, noted to pant excessively, and was frequently rotated, all of which may have facilitated migration. It is unknown whether an unrecognized congenital diaphragmatic defect or the preceding subtotal pericardiectomy contributed to the complication; however, it is suspected that the subtotal pericardiectomy had minimal influence in tube migration as the pericardium only covers the ascending aorta (8, 9). The theory that the tube may have migrated through the aortic hiatus due to initial inadvertent positioning within the mediastinum at the time of placement can conclusively be ruled out as there was both direct intraoperative visualization and immediate post-operative radiographic confirmation of appropriate tube positioning. Instead, it is suspected that intrapleural use of bupivacaine may have played a significant role in the tube migration.

Multiple controlled canine studies demonstrate that intrapleural bupivacaine provides effective postoperative analgesia while causing few adverse effects on respiratory parameters (7, 16). Some studies show evidence of superiority of intrapleural bupivacaine when compared to systemic opioids and intercostal nerve blocks. Nevertheless, local anaesthetic systemic toxicity (LAST) is a recognized risk, most often associated with dosing errors or inadvertent administration of local anaesthetic agents into perivascular, intravascular spaces, or both.

Clinical manifestations of LAST range from central nervous system excitation to CNS depression, along with severe cardiovascular effects such as arrhythmias, hypotension, bradycardia, and cardiac arrest (7, 16–18). Among local anaesthetics, lidocaine and bupivacaine are most frequently implicated in LAST, with bupivacaine posing the highest risk of dose-dependent cardiotoxicity (7, 16). Intrapleural administration of bupivacaine has also been associated with phrenic nerve dysfunction in some reports in people, potentially impairing diaphragmatic function (18). This raises the theoretical concern that impaired diaphragmatic function related to intrapleural administration of bupivacaine may reduce crural apposition and thereby reduce the structural integrity of the aortic hiatus, which may predispose thoracostomy tube migration through the aortic hiatus.

Local anaesthetic systemic toxicity (LAST) is often dose-dependent; however, it can occur at lower doses depending on the route of administration, rate of absorption, and individual patient factors (18–23). In dogs, neurotoxic effects of bupivacaine have been reported at doses of approximately 4.3–4.9 mg/kg, whereas cardiotoxic effects may occur at doses of 4–5 mg/kg, with more severe and often lethal cardiovascular toxicity reported at higher doses, 8.6–9.8 mg/kg (20). Cardiotoxicity of bupivacaine in dogs commonly manifests as bradycardia and conduction abnormalities, as the drug directly depresses myocardial contractility, disrupts calcium channel regulation, and markedly slows ventricular conduction (24). Bupivacaine is more cardiotoxic than other local anaesthetics due to its higher affinity for, and prolonged binding to, cardiac sodium channels, which further exacerbates myocardial depression (20). In this case, bradycardia was not observed; instead, tachycardia occurred. Although tachycardia with LAST is often associated with resuscitative drugs (e.g., epinephrine), this does not explain the patient’s response. Previous studies in dogs have shown that at neurotoxic doses, bupivacaine can sometimes transiently increase heart rate, likely representing an initial sympathetic response before more severe cardiovascular toxicity (20). This may account for the absence of bradycardia in our case, as the administered dose was below neurotoxic and cardiotoxic thresholds.

Regardless, when LAST occurs, early recognition and prompt intervention are indicated to improve patient survival. Management is primarily supportive and may include benzodiazepines for seizure control, oxygen supplementation, and advanced cardiac life support as indicated. In people, intravenous lipid emulsion (ILE) therapy has emerged as a critical rescue treatment in severe cases. The mechanism of action of ILE is thought to involve binding and sequestration of lipophilic drugs, redistributing these agents away from target tissues, increasing myocardial energy substrate availability, and exerting direct positive inotropic effects (19–22). Dosing protocols and indications for ILE use in veterinary patients are extrapolated from human medicine and limited animal studies. A commonly recommended regimen consists of a 20% lipid emulsion given as a 1.5–2.0 mL/kg IV bolus over 1–5 min, followed by continuous infusion at 0.25 mL/kg/min for 30–60 min, with a maximum cumulative dose of 8–10 mL/kg (23–25). Case reports in animals document rapid reversal of neurologic and cardiovascular signs of LAST following ILE administration with few adverse effects (24–28), although reported complications of ILE administration include transient hypertriglyceridemia, electrolyte abnormalities, pancreatitis, respiratory compromise, and laboratory interference (e.g., lipemia) (24). Furthermore, the physicochemical properties of bupivacaine support the use of intravenous lipid emulsion (ILE) therapy in cases of local anaesthetic systemic toxicity (LAST). Lipophilicity, commonly expressed as the octanol–water partition coefficient, or Log P, is a critical determinant of ILE efficacy, as more lipophilic compounds are preferentially sequestered into the plasma lipid phase, thereby reducing free drug concentrations in plasma and tissues (29–32). Compounds with a Log *p* > 2 are considered highly lipophilic; bupivacaine has a Log P of approximately 3.4 (29–32). This is central to the mechanism of ILE therapy for bupivacaine toxicity and supports its use in patients with LAST.

Despite our postulations, the occurrence of LAST as an explanation for this patient’s acute decompensation remains hypothetical. Other considerations include tension pneumothorax, pleural effusion, pulmonary thromboembolism, anemia, hypovolemia, cardiac decompensation, pain, and anxiety. Anemia, hypovolemia, tension pneumothorax, pleural effusion, and cardiac dysfunction were excluded via vital signs, point-of-care testing (PCV/TS, blood pressure, blood glucose), and thoracic and abdominal radiographs. Pulmonary thromboembolism could not be definitively excluded in the absence of specific diagnostics (e.g., D-dimers, angiographic computed tomography, ventilation-perfusion scans, etc.). Other limitations of this case include the absence of measured plasma bupivacaine concentrations and the lack of advanced cross-sectional imaging to further assess thoracostomy tube migration, confirm potential peritoneal involvement, and exclude diaphragmatic perforation.

Lastly, given that the diaphragm can anatomically insert along the ventral aspect of the first to third lumbar vertebrae (L1–L3) bilaterally, and that the caudal tip of the thoracostomy tube was positioned close to the mid-line and near the second lumbar vertebra (L2), it remains possible that the device was still situated within the confines of the pleural cavity, specifically at its caudodorsal extent (33). In this context, given that the pleural space and cardiac tissue lacking an intact pericardium are highly vascular, it could be postulated that bupivacaine exposure to these tissues alone may be sufficient to induce LAST, even in the absence of tube migration. However, available experimental evidence does not support the assumption that exposure to these spaces inherently results in increased systemic absorption of local anaesthetics in dogs (34). Accordingly, this would not necessarily account for the patient’s observed response to bupivacaine administration especially since the bupivacaine dose was below commonly reported neurotoxic and cardiotoxic thresholds. Whereas migration into the peritoneal or retroperitoneal space could plausibly result in a systemic effect, as systemic absorption from highly vascular abdominal sites are more rapid and extensive, leading to higher peak plasma concentrations and an increased risk of toxicity (9).

Furthermore, while some improvement may have resulted from tube removal alone, the patient remained obtunded and relatively tachycardic immediately afterward, suggesting this intervention alone was insufficient. The subsequent rapid improvement following intravenous lipid emulsion supports the hypothesis that both tube removal and lipid therapy contributed to resolution of clinical signs. Collectively, these findings support a diagnosis of local anaesthetic systemic toxicity secondary to inadvertent peritoneal and periaortic bupivacaine delivery associated with thoracostomy tube migration.

## Conclusion

This report describes the first documented case of presumed thoracostomy tube migration through the aortic hiatus into the abdominal cavity. The case further illustrates presumptive peri-aortic administration of bupivacaine resulting in local anaesthetic systemic toxicity (LAST), which was successfully managed with intravenous lipid emulsion (ILE) therapy. Although complications associated with thoracostomy tubes are uncommon, clinicians should remain aware of the potential for rare but severe adverse outcomes, particularly in overweight patients undergoing thoracic surgery and those receiving intrapleural local anaesthetics. While intrapleural bupivacaine may provide effective analgesia, vigilance for signs of systemic toxicity, including phrenic nerve dysfunction, is warranted. Continuous telemetry, monitoring of respiratory parameters, and serial thoracic imaging should be considered in patients with tube thoracostomies receiving intrapleural analgesia. Emergent repeat thoracic imaging is recommended at the first indication of clinical deterioration. Finally, ILE should be considered a viable therapeutic option for patients exhibiting clinical signs consistent with LAST following local bupivacaine administration.

## Data Availability

The original contributions presented in the study are included in the article/supplementary material, further inquiries can be directed to the corresponding author.
